# Synaptic Ensemble Underlying the Selection and Consolidation of Neuronal Circuits during Learning

**DOI:** 10.3389/fncir.2017.00012

**Published:** 2017-03-02

**Authors:** Yoshio Hoshiba, Takeyoshi Wada, Akiko Hayashi-Takagi

**Affiliations:** ^1^Laboratory of Medical Neuroscience, Institute for Molecular and Cellular Regulation, Gunma UniversityMaebashi, Japan; ^2^PRESTO, Japan Science and Technology AgencyKawaguchi, Japan

**Keywords:** synaptic ensemble, cell ensemble, dendritic spines, memory

## Abstract

Memories are crucial to the cognitive essence of who we are as human beings. Accumulating evidence has suggested that memories are stored as a subset of neurons that probably fire together in the same ensemble. Such formation of cell ensembles must meet contradictory requirements of being plastic and responsive during learning, but also stable in order to maintain the memory. Although synaptic potentiation is presumed to be the cellular substrate for this process, the link between the two remains correlational. With the application of the latest optogenetic tools, it has been possible to collect direct evidence of the contributions of synaptic potentiation in the formation and consolidation of cell ensemble in a learning task specific manner. In this review, we summarize the current view of the causative role of synaptic plasticity as the cellular mechanism underlying the encoding of memory and recalling of learned memories. In particular, we will be focusing on the latest optoprobe developed for the visualization of such “synaptic ensembles.” We further discuss how a new synaptic ensemble could contribute to the formation of cell ensembles during learning and memory. With the development and application of novel research tools in the future, studies on synaptic ensembles will pioneer new discoveries, eventually leading to a comprehensive understanding of how the brain works.

## Introduction

**Figure 1:**
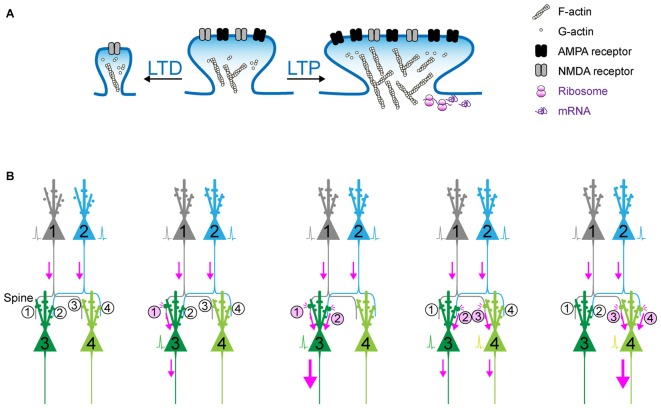
**Synaptic plasticity as the cellular basis of memory? (A)** Schematic of long-term potentiation (LTP) and long-term depression (LTD). Upon a diverse array of stimuli, the dendritic spine undergoes marked structural change, which is accompanied with the dynamic change in actin polymerization and the increase in surface AMPA receptor in the postsynaptic density (PSD). **(B)** The synaptic weights determined by (de)potentiation provide a scalar quantity representing the circuit that can be preferentially retrieved in response to the activation of upstream cell ensembles. When Spine #1 is potentiated, the probability of action potential of Neuron #3 would increase. When both Spine #1 and Spine #2 are potentiated, Neuron #3 is more likely to fire as a part of cell ensemble. In contrast, when Spine #3 is potentiated and Spine #1 is depotentiated, Neuron #4, instead of Neuron #3, is more likely to fire.

Memories provide all kinds of information and knowledge about what we have done and with whom we have interacted during the course of our lives. They are essential in creating and developing our personality, which will shape ourselves throughout life. Thus, memories are crucial to the cognitive essence of who we are as human beings. Once a memory is created, it gets stored in multiple steps including sensory recognition of an event, followed by the formation of a short-term memory (STM), and finally that of a long-term memory (LTM; McGaugh, 2000). Two characteristics distinguish STMs from LTMs: temporal decay and limited content. STMs are processed to LTMs through the process of consolidation. It is now accepted that LTMs are not stored in small regions of the brain (Lashley, 1950; Wiltgen et al., 2004), but are instead stored throughout the brain as a subset of neurons that probably fire together in the same pattern each time (Frankland and Bontempi, 2005). However, the mechanism underlying how specific subsets of neurons (cell ensembles) are selected and consolidated during information processing and learning remains to be elucidated. The phenomenon of long-term potentiation (LTP), which is operationally defined as a long-lasting increase in the synaptic efficacy upon high-frequency stimulation, has attracted great interest in the neuroscience field because of the possibility that it might underlie some aspects of learning and memory (Bliss and Collingridge, 1993). The characteristic of LTP is that it can cause long-lasting strengthening of the synapses between two neurons (Figure 1). Synaptic potentiation is associated with hippocampal LTP and is presumed to be the cellular substrate for learning and memory (Yuste and Bonhoeffer, 2001). However, owing to the lack of specific labeling probes for synaptic potentiation, it is hard to locate where the potentiated synapses are distributed throughout the brain during learning and memory processes. Furthermore, since systematic *in vivo* manipulation of specific subsets of spines has not been possible, the link between morphological modifications of synaptic spines and memory remains correlational. With the application of the latest research tools, direct evidence shows that structural plasticity of the dendritic spines is required for the recall of memory (Hayashi-Takagi et al., 2015). In this review, we summarize the current view of the cellular mechanisms underlying learning and memory, with special focus on synaptic plasticity, by which a new set of “synaptic ensembles” could contribute to the formation of “cell ensembles.”

## Cell Ensembles: The Cellular Basis of Memory?

The hypothesis that learning and memory stems from changes in the strengths of the synapses was first suggested by Cajal (1894). Cajal utilized the Golgi staining method to investigate the microscopic structure of the brain, and proposed that neurons are not physically connected to each other, but are instead separated by small spaces called synapses. While this concept, also known as the Neuron doctrine, is the central principle of modern neuroscience, it was the subject of great controversy when first proposed. Nonetheless, it is surprising that Cajal predicted over a century ago that information flows from the axon to the dendrite through the synapse, and that information storage should rely on changes in the strengths of synaptic connections. Donald Hebb, a neuropsychologist, supported and further developed Cajal’s hypothesis. At the time, the so-called Behaviorism theory flourished on the basis of the proposition that a behavior is produced either owing to reflexes produced by a response to certain stimuli, or a consequence of that individual’s history of reinforcement and punishment (Watson, 1913). In the same period, a largely distinct theory, the Gestalt psychology/Field theory, was also influential (Lewin, 1939). This theory assumed that behavior is too varied and complex for it to be explainable as the sum of parts, and brain function is due to spreading and interacting fields of electrical activity. In 1949, Hebb presented his own behavioral observations made from experimental rats and human patients, and reconciled these two distinct principles, proposing Hebb’s Postulate which can be stated as follows: *“When an axon of cell A is near enough to excite cell B and repeatedly or persistently takes part in firing it, some growth process or metabolic change takes place in one or both cells such that A’s efficiency, as one of the cells firing, is increased.”* This was paraphrased as *“neurons that fire together, wire together”* by Lowel and Singer (1992). Furthermore, Hebb theoretically developed the concept of “cell ensemble” that is defined as *“a diffuse structure comprising cells in the cortex and diencephalon, capable of acting briefly as a closed system, delivering facilitation to other such systems. Depending on functional requirements, individual cells could participate in different cell assemblies and be involved in multiple computations.”* It is noteworthy that when Hebb published this principle, the synapse had still not been visualized, but his speculative description of the “Hebb synapse” and its properties continued to greatly impact neuroscience (Brown and Milner, 2003). One problem with demonstrating this hypothesis was that it was difficult to directly record the activity of single synapses in a behaving animal. Thus, the challenge in the field has been relating changes in synaptic efficacy to specific behavioral learning. Considerable effort has been dedicated to understand the mechanism by which strengthening of synaptic connections can be achieved. One model that fulfills Hebb’s plasticity is that of LTP in the rabbit hippocampus, which was fully described by neurophysiologists Terje Lømo and Timothy Bliss in 1973 (Bliss and Gardner-Medwin, 1973; Bliss and Lømo, 1973). LTP is defined as a long-lasting increase in synaptic efficacy following high frequency bursts of electrical stimulation (Figure 1); it is now demonstrated that many forms of LTP, Hebbian and non-Hebbian potentiation, occur in various other brain regions as well (Bliss and Collingridge, 1993; Maren, 1999). LTP has since become the primary experimental model of learning and memory based on the experimental evidence that hippocampus-dependent learning is accompanied with hippocampal LTP, and that it produces the similar increases in glutamate receptors, amplitudes of evoked synaptic transmission and neuronal responsiveness (Skelton et al., 1987; Whitlock et al., 2006). Since learning-induced synaptic potentiation occluded high-frequency stimulation-induced LTP, it can be concluded that hippocampus-dependent learning induces LTP in the hippocampus (Whitlock et al., 2006). Consistently, pretreatment of LTP stimulation just before learning acquisition was seen to disrupt subsequent spatial learning, while it did not affect well-established spatial memories. As the authors suggested, this could be the result of LTP exhausting the entire population of modifiable synapses (McNaughton et al., 1986). Pharmacological intervention, such as chronic intraventricular infusion of an N-Methyl-D-aspartate (NMDA) receptor antagonist, prevents the induction of both, hippocampal LTP and hippocampus-dependent place learning (Morris et al., 1986). Owing to these marked associations between LTP and learning, LTP and other types of synaptic plasticity have attracted considerable interest as one of the major cellular mechanisms underlying learning and memory. The next challenging question is how sensory information during learning is processed and integrated into neuronal circuits as synaptic changes, and how this eventually influences the behavioral output. The simple invertebrate model such as Aplysia have provided a powerful strategy to comprehensively address this question, owing to their simple nervous systems. In Aplysia, some of the neurons are giant, measuring approximately 1 mm in diameter. Owing to this simplicity and large cell sizes, each cell can be subjected to electrophysiological assessment in the presence of versatile molecular and pharmacological interventions, and the same cell can be repeatedly recorded from over a period of days, and subsequent biochemical analyses. By utilizing this elegant system, Kandel (2001) have revealed that many forms of synaptic plasticity, which differ in their mechanism of induction, are associated with simple memory processes such as habituation, sensitization and classical conditioning. Furthermore, recent advances in *in vivo* imaging and recording techniques have enabled us to study the relationship of neuronal activity and subsequent plasticity with various behavioral changes in rodents and non-human primates (Komiyama et al., 2010; Scott et al., 2013; Sadakane et al., 2015). For instance, a combination of genetically encoded calcium indicators and *in vivo* two-photon imaging can show the longitudinal trajectory of spatial patterns of neuronal activity in behaving animals, showing that neural activities in the motor cortex changed with motor learning (Komiyama et al., 2010; Masamizu et al., 2014; Peters et al., 2014). Recent *in vivo* electrophysiological techniques using tetrodes have also shown a correlation between cell population activity and specific experiences (Davidson et al., 2009). A recent study demonstrated that repetitive two-photon stimulation of selective neuronal populations generated artificial cell ensembles that spontaneously recur (Carrillo-Reid et al., 2016), which is consistent with the hypothesis that Hebbian plasticity contributes to the formation of cell ensembles. Since the existence and properties of cell ensembles have been suggested, the next challenge is to demonstrate the causal relationship between cell ensembles and specific behaviors. In spite of a vast body of elegant scientific works showing correlations between cell ensembles and learning, the causal link between the two has been difficult to show. However, the emergence of versatile and state-of-the-art techniques for neuronal manipulation such as optogenetics and pharmacogenetics has offered new tools and opportunities for finding a definitive and causal answer for the great pioneers’ century-old question: are changes in cell ensembles via synaptic plasticity a possible mechanism underlying learning and memory?

## State-of-the-Art Techniques for the Manipulation of Cell Ensembles

Pharmacogenetic approaches, such as designer receptor exclusively activated by designer drugs (DREADDs), are currently available to control neuronal firing. DREADDs are engineered G-protein coupled receptors that are activated by otherwise inert drug-like small and diffusible ligands. Gq-coupled DREADD induces neuronal firing, while Gi-coupled DREADD mediates neuronal and synaptic silencing, thereby allowing non-invasive and broad modulation of neuronal firing (Armbruster et al., 2007; Alexander et al., 2009; Stachniak et al., 2014). Optogenetics is another powerful technique that stems from the fruitful fusion of optics and genetic engineering methods, maximizing the advantages of each discipline: regulation of optical control with light of desirable wavelength and intensity in the millisecond time scale, and specific gene expression and trafficking of the gene product of interest with subcellular precision (Lima and Miesenböck, 2005; Ishizuka et al., 2006; Boyden, 2011; Deisseroth, 2015). The rediscovery and optimization of microbial opsins, such as channelrhodopsins-2 (ChR2), as light-activated cation channels has enabled the control of the membrane potential of targeted neurons, driving it above the action potential threshold, and resulting in the ability to control neural activity with blue light with millisecond-scale temporal precision (Boyden et al., 2005). In addition, photoinhibitory membrane proteins such as archaerhodopsin-3 (Arch) have been discovered. They are green-yellow light-driven outward proton pumps mediating rapid and reversible silencing of neural activity by pumping protons out of a cell, thus hyperpolarizing it (Chow et al., 2010). Therefore, optical activation, silencing and (de)synchronization of neuronal activity have now become possible. Until recently, much of the scientific evidence supporting the involvement of synaptic plasticity in the memory formation process was based on a correlation between variables—that is the parallel occurrence between a factor and a phenomenon—which does not indicate any causality in the relationship. In addition to correlation, necessity and sufficiency are also critical components of scientific reasoning that establish causation. A factor is considered necessary if it is an absolute requirement for the phenomenon. In the biological context, if manipulation of the factor interferes with the phenomenon, the factor is necessary for the expression of the phenomenon itself. The factor is sufficient if it can produce the phenomenon without the addition of any other factors. It is also considered sufficient if an artificial execution of such a factor elicits the phenomenon. In this regard, the emergence of optogenetics/pharmacogenetics provides a completely new way to provide evidence at the level of necessity and sufficiency in order to elucidate how neuronal networks work, and how they relate to learning and memory, while also providing important information about neuronal coding and computation of neuronal circuits (Tye and Deisseroth, 2012; Herry and Johansen, 2014). Numerous elegant studies utilizing these techniques for the dissection of neuronal circuits have been published and reviewed in detail elsewhere (Carter and de Lecea, 2011; Urban and Roth, 2015). Therefore, we will focus on a limited number of representative studies such as those involving fear memory.

Lesion studies support a role for the amygdala in the storage of fear memories. The amygdala is a complex of more than ten nuclei with distinct connections within the intra-amygdala nuclei as well as those with an array of neocortical areas (Sah et al., 2003). In addition, the amygdala has strong connections with the autonomic nervous system, receiving inputs from all the sensory modalities as well as visceral inputs. The amygdala strongly regulates emotional responses, attention, perception and memory of danger (Phelps and LeDoux, 2005). To date, what amygdaloid pathway is responsible for emotional memory formation remains unknown. Optical activation of ChR2 in the specific pathways from the basolateral amygdala (BLA) to the central nucleus of the amygdala (BLA-CeA pathway) and to the nucleus accumbens (BLA-NAc pathway) was shown to elicit opposing synaptic changes following reward or fear learning, respectively (Namburi et al., 2015). This work elegantly dissected how synaptic potentiation in specific neuronal circuits in the BLA can underlie learned associations that lead to such different reward-related and fear-related behavioral outputs. Another excellent example comes from the Tonegawa Lab (Liu et al., 2012). The hippocampus is reported to be critical for the formation of contextual fear memories. Therefore, they expressed ChR2 in an activity-dependent and doxycycline-regulated manner in the hippocampus, allowing only neurons activated during a conditioning period to become labeled by ChR2 and re-activated by blue light. In other words, this manipulation directly examined the hypothesis that the specific subset of hippocampal neurons activated during conditioning are sufficient for memory recall. Indeed, optical re-activation of these cells resulted in freezing behavior indicative of fear memory, thus demonstrating the causal contribution of these conditioning-activated cell ensembles to the memory. Consistent with this finding, the Mayford lab (Garner et al., 2012) demonstrated that the pharmacogenetic activation of Gq-coupled DREADD-expressing cells, which were labeled during conditioning, was required for memory retrieval. Conversely, reactivation of the conditioning-unrelated network impaired memory retrieval. These findings clearly suggest that the activation of a learning-related cell ensemble can artificially generate a synthetic memory.

It should be noted that the conventional memory research is sometimes compromised by data interpretation: when we train an animal under different sets of conditions, the readout we can measure is a behavioral phenotype measured at a later time point. In this case, if an animal exhibits memory deficits under a certain condition, it is sometimes difficult to draw a conclusion about what process, whether of encoding, consolidation, or recall, is disrupted. The Tonegawa group overcame this limitation by combining the pharmacological amnesia model with the optical activation of conditioning-activated cell ensembles (Ryan et al., 2015). In this series of experiments, a moderate dose of the protein synthesis inhibitor, anisomycin, was injected into mice immediately after contextual fear conditioning. This dose of anisomycin disrupted the protein synthesis-dependent synaptic potentiation, but allowed the activity-dependent labeling of ChR2 during the conditioning period. This experimental design apparently prevented learning-dependent synaptic potentiation, and further induced amnesia corresponding to the conditioning. Nonetheless, direct optical activation of ChR2-positive neurons that were labeled during conditioning resulted in memory retrieval, which suggests that synaptic potentiation is not a crucial component of memory recall as long as a proper subset of cell ensembles can be optically activated. However, in this experimental design the apparent amnesia under the block of synaptic potentiation suggested that conditioning-elicited synaptic potentiation can provide natural recall cues that drive an efficient level of activation of conditioning-related cell ensembles. Consistently, an artificial memory could be disrupted by synaptic depression (Nabavi et al., 2014). In this sense, synaptic potentiation is crucial during encoding and recall phases. Synaptic (de)potentiation can actively change the strength of connectivity in neuronal circuits, resulting in the selection and consolidation of cell ensembles from a diverse group of neurons distributed across multiple brain regions. Synaptic connectivity can hold the memory specificity because the synaptic weights determined by (de)potentiation provide a scalar quantity representing the circuit that can be preferentially retrieved in response to the activation of upstream cell ensembles (Figure 1B). This is one of the reasons why synapses are considered the basic units of memory.

## Synaptic Ensembles as Unitary Bases of Cell Ensembles

**Figure 2:**
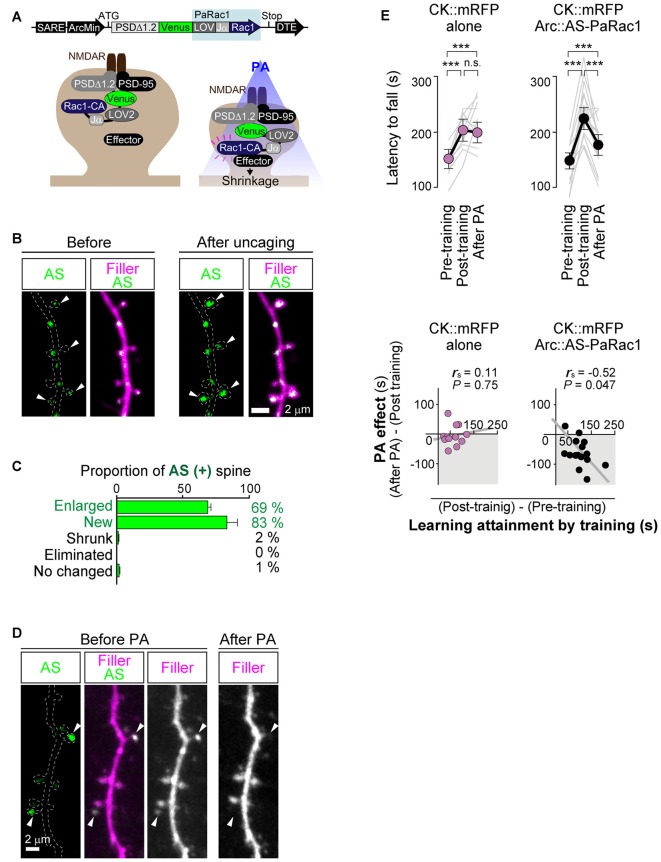
**Selective labeling and shrinkage of potentiated synapses by AS-PaRac1. (A)** Schematic representation of the AS-PaRac1 vector, which was transcribed under the control of synaptic activity-responsive element (SARE) and the *Arc* minimal promoter. The LOV2 domain was attached to the N terminus of the constitutively active form of Rac1 (Rac1-CA), blocking the effector binding site of Rac1 until the blue light irradiation led to the unwinding of the LOV Jα helix. Therefore, Rac1 activity could be controlled by blue light, enabling optical control of spine shrinkage. **(B)** Protein synthesis-dependent potentiation during the single spine LTP protocol, which was elicited by glutamate uncaging in the presence of the adenylyl cyclase activator forskolin, induced the accumulation of AS-PaRac1 in the stimulated spines (arrowheads). Hippocampal slice culture (DIV 11). **(C)** AS-PaRac1 labeling *in vivo* during the rotarod task. Percentage of AS-PaRac1-containing spines for enlarged (Δ spine volume ≥ 50%), newly formed, shrunk (Δ spine volume ≥ −50%) and eliminated spines following the rotarod task. **(D)** Selective shrinkage of AS-PaRac1-positive spines by photoactivation (PA). Neurons in the hippocampal slice culture (DIV 11) were biolistically transfected with AS-PaRac1-Venus and filler. Robust shrinkage (arrowheads) was observed, while AS-PaRac1-negative spines were not affected by PA, despite being located adjacent to the AS-PaRac1-positive spines. **(E)** Erasure of the acquired learning by PA of the potentiated spines labeled with AS-PaRac1. The mice were divided into two groups: animals in the first group were infected with CaMKII promoter::mRFP (CK::mRFP) alone as a control, while those in the second group were infected with AS-PaRac1 and mRFP in the primary motor cortex (M1). Both the groups exhibited significantly better motor performance after training (Post-training), but only the performance of the AS-PaRac1 group was disturbed by PA (After PA), and the extent of learning disruption induced by PA (PA effect) negatively correlated with the extent of training-evoked improvement (learning attainment). In contrast, there was neither a disruption of acquired learning nor a correlation between the effects of training and of PA in the control group. One-way repeated measures ANOVA for the comparison of task performance for the same subjects at different time points followed by *post hoc* Scheffé’s test (***P < 0.001) and Spearman’s rank correlation were used. Abbreviations: AS-PaRac1, Activated Synapse-targeting PhotoActivatable Rac1; DIV, days *in vitro*; LTP, long-term potentiation; CaMKII, Calcium/calmodulin-dependent protein kinase II; mRFP, monomer red fluorescent protein; ANOVA, analysis of variance. Image adapted, with permission, from Hayashi-Takagi et al. (2015).

**Figure 3:**
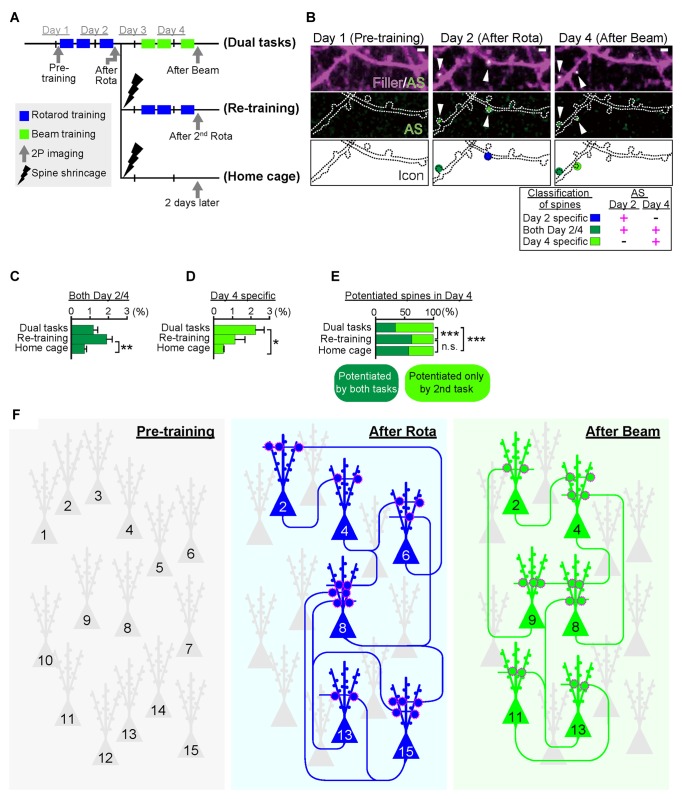
**Task-specific synaptic ensembles. (A)** Experimental design. Layer II/III pyramidal neurons in the M1 were sparsely transduced with Arc::AS-PaRac1-Venus and CAG::mRFP (filler). Mice were subjected to distinct training protocols. In the dual task protocol, mice sequentially learned two distinct hindlimb tasks: the rotarod and beam tasks in the first and the latter 2 days, respectively. The re-training protocol group was subjected to the rotarod task in the first 2 days, which was followed by the shrinkage of the learning-evoked potentiation by PA, and then the identical rotarod task was re-trained. The homecage group was subjected to the rotarod task and subsequent PA, and mice were not touched or trained for another 2 days. **(B)** Representative images of dendrites that exhibited potentiation during learning. AS-PaRac1 puncta are color-coded based on the appearance and duration of fluorescence: the rotarod task potentiation (day 2-specific, blue), beam task-specific potentiation (day 4-specific, greenish yellow), and continuous potentiation for both periods (both day 2 and 4, dark green). **(C)** Significant increase was seen in the potentiation on both days 2 and 4 in the re-training group, suggesting that re-training induced re-potentiation of the same subset of spines. **(D,E)** The proportion of newly potentiated spines, which had not been potentiated in the first 2 days, significantly increased only in the dual task group. **(F)** Conceptual comparison between cell ensembles and synaptic ensembles. The schema was depicted based on our findings that the cell ensembles for the rotarod and beam tasks was significantly more overlapping than the synaptic ensembles. The difference in pattern between cell ensembles and synaptic ensembles supports the importance of visualization of synaptic ensembles as well as that of cell ensembles to understand how the brain is reorganized during learning and memory. Statistical significance was tested with ANOVA followed by *post hoc* Scheffé’s test for **(C, D)**, and Chi-square test was used to test independence for **(E)**. For all statistical test *P < 0.05, **P < 0.01, ***P < 0.001 were considered significant. n.s., not significant. Abbreviations: AS-PaRac1, Activated Synapse-targeting PhotoActivatable Rac1; mRFP, monomeric red fluorescent protein; ANOVA, analysis of variance. Images from **(A–E)**, adapted, with permission, from Hayashi-Takagi et al. (2015).

As mentioned above, Cajal predicted over a century ago that memory must rely on changes in the strengths of synaptic connections. In the mammalian neocortex, a majority of excitatory synapses is formed onto a dendritic spine, a small protrusion of the dendrite. Recent advances in *in vitro* as well as *in vivo* brain imaging techniques have allowed the direct visualization of dendritic spines and the plasticity of their structure; many neuroscientists have been attracted by the drastic plasticity of the spine structure for decades (Bhatt et al., 2009; Holtmaat and Svoboda, 2009; Kasai et al., 2010; Yuste, 2011). Spines can undergo rapid enlargement or grow *de novo* in response to LTP-inducing stimuli, such as electrical stimulation (Engert and Bonhoeffer, 1999), two-photon glutamate uncaging (Matsuzaki et al., 2004), optogenetic stimulation (Fuchikami et al., 2015), or altered sensory experiences (Trachtenberg et al., 2002; Zuo et al., 2005). In sharp contrast, long-term depression (LTD)-inducing stimuli lead to shrinkage of the spine head and increased spine loss (Wiegert and Oertner, 2013). There is a strong positive correlation among the spine head size, the postsynaptic density (PSD) area, the presynaptic active zone area, and the amplitude of AMPA receptor-mediated excitatory postsynaptic currents (EPSCs) recorded in the spine (Matsuzaki et al., 2001; Shapira et al., 2003; Arellano et al., 2007; Bourne and Harris, 2008). Because as rapid and selective enlargement of stimulated spines in response to glutamate uncaging is associated with an increase in the amplitude of AMPA receptor-mediated currents selectively at the stimulated synapse but not with those at the neighboring spines (Matsuzaki et al., 2004), these findings strongly suggested that the spine structure is tightly coupled to synaptic function. A recent study using *in vivo* two-photon laser scanning imaging through a cranial window revealed that dendritic spines are very plastic and motile even in the adult brain, and are significantly regulated by various paradigms such as sensory deprivation or motor skill learning (Zuo et al., 2005; Holtmaat and Svoboda, 2009). Importantly, the extent of spine formation is closely associated with that of learning acquisition and maintenance of the skill (Xu et al., 2009; Yang et al., 2009). These studies suggest a critical role of spine reorganization in the formation of motor memories. Nonetheless, clear visualization of the spine structure *in vivo* requires the sparse labeling of neurons; for example, a frequently used mouse strain, Thy1-EGFP-M line, expresses enhanced green fluorescent protein (EGFP) in sparse subsets of neurons only in the layer V pyramidal neurons (Feng et al., 2000). This hinders the comprehensive analysis of how the spine structural changes would be distributed throughout the brain. Furthermore, despite the significant correlation between spine dynamics and learning behavior, their causal relationship remains unclear because of the lack of established techniques for manipulating individual synapses *in vivo*. To overcome this problem, we recently developed a novel synaptic optoprobe, AS-PaRac1 (Activated Synapse-targeting PhotoActivatable Rac1), which can specifically label recently potentiated (enlarged or newly formed) spines in a transcription- and translation-dependent manner (Hayashi-Takagi et al., 2015). AS-PaRac1 is a fusion protein that consists of PSD-95, Venus, photoactivatable Rac1 (PaRac1; Wu et al., 2009), and the 5′ and 3′ untranslated regions (UTRs) of the *Arc* gene (Figure 2A). The mechanism by which AS-PaRac1 selectively labels the potentiated spines (Figures 2B,C) has been described in detail elsewhere (Extended Data Figure 3 from Hayashi-Takagi et al., 2015); briefly, it takes advantage of the N-terminal of PSD-95 and the dendritic targeting element (DTE) in the 3′ UTR of the *Arc* mRNA. The *Arc* mRNA is targeted to stimulated dendritic segments in an activity-dependent manner (Steward and Worley, 2001), and the DTE is known to be crucial for this targeting (Kobayashi et al., 2005). This means that the *AS-PaRac1* mRNA is transcribed in an activity-dependent manner like the *Arc* mRNA, followed by its targeting to the dendritic segment with active synaptic input. Importantly, because the majority of the AS-PaRac1 protein is derived from PSD-95, the AS-PaRac1 protein behaves like the PSD-95 proteins after the activity-dependent local translation of the *AS-PaRac1* mRNA. The potentiated synaptic spine rapidly and markedly increases the PSD area, capturing diffusing PSD-95 proteins far more efficiently than small and stable spines (Gray et al., 2006). Once the AS-PaRac1 protein is integrated into the PSD, the ubiquitination site on the N-terminal of the AS-PaRac1 protein is presumably concealed, thus rendering it relatively stable. Thus, *in vivo* mapping of synaptic potentiation now becomes possible. Mice that were transduced with AS-PaRac1 in the primary motor cortex (M1) were subjected to a hindlimb-related motor skill test. We found that this evoked substantial remodeling of spines (14.7 ± 2.01%) in a small neuronal population (16.4 ± 2.8%) in the layer II/III. Similarly, we observed spine remodeling (5.01 ± 0.76%) in a small population of layer V neurons (22.6 ± 2.8%) in response to learning. A distinctive property of AS-PaRac1 is that it not only specifically labels recently potentiated spines, but also induces the shrinkage of spines upon blue light, indicating that blue light can specifically elicit the shrinkage of the recently potentiated spines (Figure 2D). Indeed, the acquired rotarod motor learning was disrupted by blue light (Figure 2E), whereas it was not affected by the identical manipulation of spines evoked by a distinct motor task in the same cortical region. Direct visualization of synaptic ensembles during the distinct hindlimb task (rotarod and beam tasks; Figures 3A,B) revealed that more than half of the beam-evoked potentiation were new potentiation, which were not potentiated previously in the rotarod task (Figure 3E). In contrast, re-training with the identical task induced the re-potentiation of the same subset of spines (Figure 3C). Taken together with the behavioral data, these findings suggest that these different learning tasks induced the potentiation of distinct synaptic ensembles (Figures 3D–F).

## Future Direction for Research for Synaptic Ensemble

**Figure 4:**
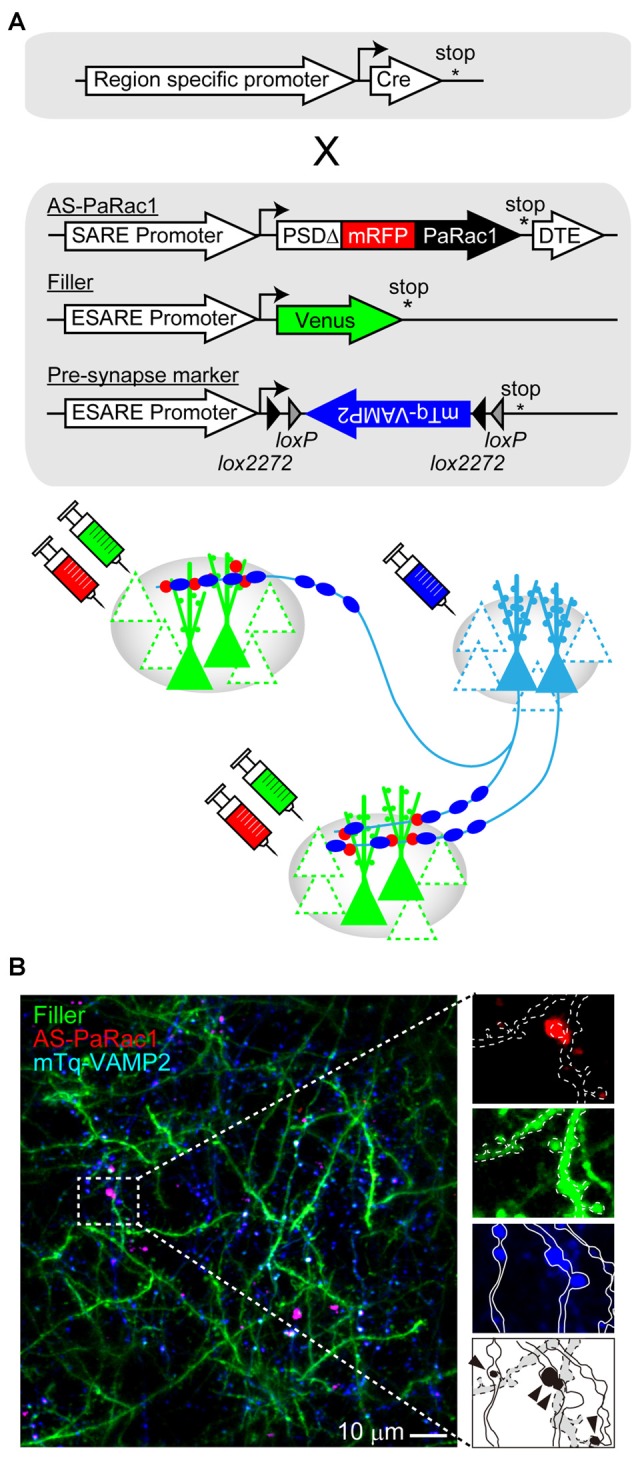
**Challenges for simultaneous visualization of both presynaptic and postsynaptic activation. (A)** Construct design. To visualize the synchrous firing of the presynaptic and the postsynaptic neurons, AS-PaRac1, neuronal filler, as well as presynaptic marker VAMP2-fused with mTurquoise (mTq-VAMP2) were transduced. For the selective labeling of the circuit of interest, CRE-dependent expression of the presynaptic marker (doublefloxed inverted open-reading-frame, DIO) was used. **(B)** Representative image of the simultaneous visualization of presynaptic and postsynaptic activation. The merged puncta (labeled by both, mTq and mRFP) are supposed to be Hebbian synaptic plasticity (arrowheads). Abbreviations: AS-PaRac1, Activated Synapse-targeting PhotoActivatable Rac1; mRFP, monomeric red fluorescent protein for visualization of neuronal morphology; VAMP2, vesicle-associated membrane protein 2.

As mentioned in the previous section, AS-PaRac1 is a new and unique optoprobe that can help in the elucidation of the dynamic formation of synaptic ensembles in live animals. In this section, we will propose further potential applications of this technique. Among the many forms of synaptic plasticity, Hebbian plasticity is an important cellular mechanism that is often used to model activity-dependent reorganization of neuronal selectivity to various aspects of learning (Brown and Milner, 2003). This mechanism tends to increase post-synaptic firing rates excessively so that the neuron in the selected cell ensemble becomes more responsive to the activities of other neurons. Because the induction of Hebbian plasticity requires correlated firing of the presynaptic and the postsynaptic neurons, simultaneous visualization of both presynaptic and postsynaptic activations would provide additional insight into the mechanisms underlying synaptic plasticity. For example, potentiated presynapses can be labeled with the vesicle-associated membrane protein 2 (VAMP2) that is fused with mTurquoise. The labeling probes for both, the presynapse (mTurquoise-VAMP2) and postsynapse (AS-PaRac1), are expressed in an activity-dependent manner, and these probes can be designed for rapid decay due to the destabilizing fusion signal; this simultaneous labeling could monitor Hebbian synaptic plasticity (Figure 4). One configuration is a conditional expression of a presynaptic marker with use of a double-floxed inverted open reading frame (DIO) and Cre recombinase. This projection pathway-specific optogenetics will be helpful in order to elucidate the dynamic nature of synaptic potentiation in a specific neuronal circuit chosen from the vast complex brain. In addition, a combination of tissue clearing and light-sheet microscopy (Hama et al., 2011; Ertürk et al., 2012; Ke et al., 2013; Susaki et al., 2014; Yang et al., 2014; Keller and Ahrens, 2015; Lerner et al., 2015) will allow researchers to perform a comprehensive analysis of how such potentiation is distributed throughout the brain at a single-synapse resolution.

## Concluding Remarks

In this review, we summarized the evidence about how memories are encoded and recalled in the form of synchronous activation of cell ensembles. Cell ensembles are organized by synaptic ensembles, which can allow the brain to form an astronomical number of connections. A simple organism like *C. elegans* has 302 neurons and about 5000 chemical synapses (de Bono and Maricq, 2005). Despite its simplicity, this nervous system regulates a diverse variety of behaviors such as mechano- and chemosensory responses, olfactory behaviors, locomotion, feeding and sex-specific behaviors such as egg-laying and mating (de Bono and Maricq, 2005). It has been proposed that the defined circuits responsible for each behavior connect with one another in order to produce executional hierarchies. For example, when food availability is limited, sex-specific behavior is suppressed. When a starved animal encounters food, locomotion behavior becomes suppressed, allowing the animal to feed properly. All these behaviors are plastic and are, therefore, subject to change through learning and memory. Only 302 neurons and ~5000 chemical synapses can display such a diverse array of behaviors by driving the right cell ensembles. In the adult human brain, it is estimated that there are approximately 86 billion neurons, each having hundreds to thousands of synapses, making the estimated number of functional synapses of the order of trillions (Tang et al., 2001). Innovations in genetic, imaging and electrophysiological technologies, as well as the development of new optoprobes such as AS-PaRac1 would accelerate the *in vivo* visualization and recording of dynamic processes of synapses with unprecedented resolution. As these powerful tools are becoming available, more and more studies are focusing on understanding how synaptic ensembles work, pioneering new discoveries in neuroscience research, and eventually leading to a comprehensive understanding of how the brain works.

## Author Contributions

AH-T and YH wrote the article, and TW assisted with figure preparation.

## Conflict of Interest Statement

The authors declare that the research was conducted in the absence of any commercial or financial relationships that could be construed as a potential conflict of interest.
